# Systemic Inflammation in Young Adults Is Associated with Abnormal Lung Function in Middle Age

**DOI:** 10.1371/journal.pone.0011431

**Published:** 2010-07-02

**Authors:** Ravi Kalhan, Betty T. Tran, Laura A. Colangelo, Sharon R. Rosenberg, Kiang Liu, Bharat Thyagarajan, David R. Jacobs, Lewis J. Smith

**Affiliations:** 1 Asthma-Chronic Obstructive Pulmonary Disease (COPD) Program, Division of Pulmonary and Critical Care Medicine, Northwestern University Feinberg School of Medicine, Chicago, Illinois, United States of America; 2 Division of Pulmonary and Critical Care, University of Washington, Seattle, Washington, United States of America; 3 Department of Preventive Medicine, Northwestern University Feinberg School of Medicine, Chicago, Illinois, United States of America; 4 Department of Laboratory Medicine and Pathology, University of Minnesota Medical School, Minneapolis, Minnesota, United States of America; 5 Division of Epidemiology and Community Health, University of Minnesota School of Public Health, Minneapolis, Minnesota, United States of America; 6 Department of Nutrition, University of Oslo, Oslo, Norway; Erasmus University Rotterdam, Netherlands

## Abstract

**Background:**

Systemic inflammation is associated with reduced lung function in both healthy individuals and those with chronic obstructive pulmonary disease (COPD). Whether systemic inflammation in healthy young adults is associated with future impairment in lung health is uncertain.

**Methodology/Principal Findings:**

We evaluated the association between plasma fibrinogen and C-reactive protein (CRP) in young adults and lung function in the Coronary Artery Risk Development in Young Adults cohort study. Higher year 7 fibrinogen was associated with greater loss of forced vital capacity (FVC) between years 5 and 20 (439 mL in quartile 4 vs. 398 mL in quartile 1, P<0.001) and forced expiratory volume in 1 second (FEV_1_) (487 mL in quartile 4 vs. 446 mL in quartile 1, P<0.001) independent of cigarette smoking, body habitus, baseline lung function and demographic factors. Higher year 7 CRP was also associated with both greater loss of FVC (455 mL in quartile 4 vs. 390 mL in quartile 1, P<0.001) and FEV_1_ (491 mL in quartile 4 vs. 442 mL in quartile 1, P = 0.001). Higher year 7 fibrinogen and CRP were associated with abnormal FVC at year 20 (odds ratio (OR) per standard deviation 1.51 (95% confidence interval (CI): 1.30–1.75) for fibrinogen and 1.35 (95% CI: 1.14–1.59) for CRP). Higher year 5 fibrinogen was additionally associated with abnormal FEV_1_. A positive interaction was observed between pack-years cigarette smoking and year 7 CRP for the COPD endpoint, and among participants with greater than 10 pack-years of cigarette exposure, year 7 CRP was associated with greater odds of COPD at year 20 (OR per standard deviation 1.53 (95% CI: 1.08–2.16).

**Conclusion/Significance:**

Systemic inflammation in young adults is associated with abnormal lung function in middle age. In particular, elevated CRP may identify vulnerability to COPD among individuals who smoke.

**Trial Registration:**

ClinicalTrials.gov NCT00005130

## Introduction

Among both healthy individuals and chronic obstructive pulmonary disease (COPD) patients, an inverse association between blood levels of fibrinogen and C-reactive protein (CRP) and forced expiratory volume in 1 second (FEV_1_) has been reported [1], [2], [3], [4]. Recent data indicate that CRP and other inflammation-sensitive proteins may be risk factors for COPD [5], [6]. However, conflicting information exists regarding the relationship between systemic inflammation and lung function over time in young adults, and the available data are somewhat limited by small sample size [7], losses to follow-up [8], and short follow-up periods [1], [9]. Data from the Odense Schoolchild Study recently demonstrated that higher levels of CRP at age 20 are associated with greater reduction in FEV_1_ and FVC over nine years, but the young age of the study population makes it difficult to ascertain risk of future lung disease [10].

Extending our prior work which examined the short-term relationship between fibrinogen and lung function over 5 years [9], we explored the relationship between both CRP and fibrinogen measured in young adults aged 23 to 37 years and lung function measured over 15 years of followup in the Coronary Artery Risk Development in Young Adults (CARDIA) cohort study. We hypothesized that in young adults without lung disease, elevated levels of CRP and fibrinogen in young adulthood would be associated with accelerated decline in lung function over 15 years of follow-up as well as greater risk of COPD in middle age. This hypothesis, if confirmed, would imply that young adults with evidence of systemic inflammation are at increased risk for future impairments in lung health.

## Materials and Methods

### Ethics statement

All CARDIA exams were approved by the institutional review board at each site, and written informed consent was obtained from all subjects.

### Study population

**Figure 1:**
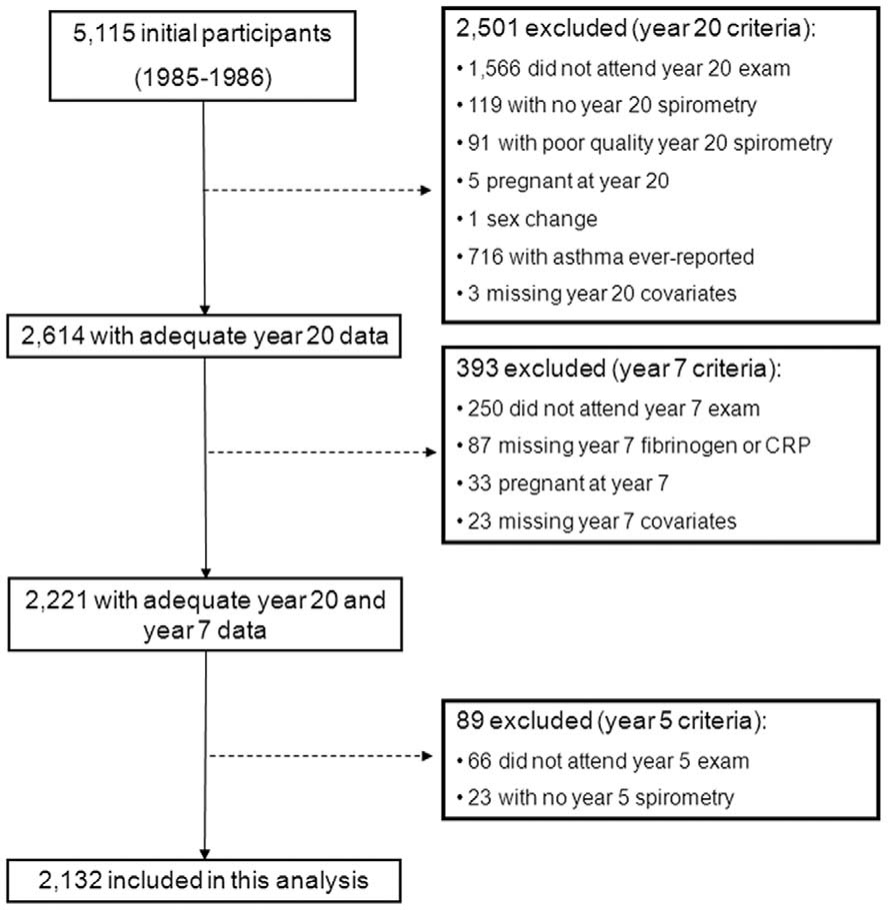
Summary of the population from CARDIA included in the present analysis.

The CARDIA Study is a cohort study of 5,115 participants conducted in 4 U.S. cities [11]. The cohort has completed a total of seven examinations: year 0 (1985–1986), year 2 (1987–1988), year 5 (1990–1991), year 7 (1992–1993), year 10 (1995–1996), year 15 (2000–2001), and year 20 (2005–2006). At the time of enrollment, virtually all CARDIA participants were generally healthy and free of any overt uncontrolled chronic medical conditions [12]. 3,549 participants were examined in year 20 (a retention rate of 72%). Participants were excluded from this analysis if they reported asthma, were on asthma medication at any of the exams, had missing or poor quality spirometry at year 20, missing CRP or fibrinogen at year 7, if pregnant at years 5, 7, or 20, or if they were transgender. The exclusions are detailed in Figure 1. The baseline characteristics of the 2,132 participants who were included in this analysis were compared to the 2,983 who either did not attend the year 20 examination or were excluded from the current analysis. Compared to those who were excluded, those included were older (25.2 vs. 24.6 years), smoked less (2.0 vs. 2.4 pack years), had lower BMI (24.2 vs. 24.7 kg/m^2^), more education (14.2 vs. 13.4 years), and higher FEV_1_ (99.2% vs. 96.8% predicted), FVC (101.0% vs. 100.0% predicted), and FEV_1_/FVC (83.4% vs. 82.7%). Excluded participants were 60% Black.

### Laboratory measurements

The earliest time-point in CARDIA at which both fibrinogen and CRP were measured is year 7. Fibrinogen was measured by a BNII nephelometer (Dade Behring, Deerfield, IL). The intra- and inter-assay coefficients of variation were 2.7 and 2.6% [13]. CRP was measured using a BNII nephelometer (Dade Behring, Deerfield, IL), with intra- and inter-assay coefficients of variation ranging from 2.3% to 4.4%, and 2.1% to 5.7% [14].

### Spirometry

Spirometry was performed at all exams except years 7 and 15. Spirometry through year 10 was measured using a Collins Survey 8-liter water sealed spirometer and an Eagle II Microprocessor (Warren E. Collins, Inc., Braintree, MA). At year 20, a dry rolling-seal OMI spirometer (Viasys, Loma Linda, CA) was used. The accuracy of each spirometer was validated using the Pulmonary Waveform Generator (MH Custom Design and Manufacturing, Midvale, UT). A comparability study performed on 25 human volunteers who performed forced vital capacity maneuvers on both spirometers demonstrated an average difference between the Collins and OMI spirometers of 6 ml for forced vital capacity (FVC) and 21 ml for (forced expiratory volume in 1 second) FEV_1_. Standard quality control and testing procedures were maintained according to American Thoracic Society (ATS)/European Respiratory Society (ERS) guidelines [15], [16], [17], [18].

### Statistical analysis

Analysis was performed in both quartiles of fibrinogen or CRP as well as with continuous data. Because the distribution of both CRP and fibrinogen differ according to race and sex, year 7 fibrinogen and CRP levels were divided into race- and sex-specific quartiles (Table 1).

**Table 1:** Characteristics of the subjects included in this analysis at the time of fibrinogen and CRP (year 7) measurement.^a^

Characteristic	Fibrinogen Quartile (year 7)				CRP Quartile (year 7)			
	1	2	3	4	1	2	3	4
Sex- and race-specific range (mean)*								
African American men	195–278 (253)	279–315 (299)	316–360 (337)	361–811 (418)	0.15–0.47 (0.30)	0.48–0.98 (0.70)	0.99–2.45 (1.60)	2.48–67.60 (6.37)
Caucasian men	173–266 (241)	267–296 (282)	297–336 (315)	337–582 (382)	0.15–0.38 (0.23)	0.39–0.76 (0.56)	0.77–1.83 (1.14)	1.86–23.60 (4.61)
African American women	107–306 (270)	307–360 (335)	361–417 (387)	418–677 (479)	0.15–0.63 (0.35)	0.64–1.77 (1.12)	1.78–4.64 (3.00)	4.65–333.00 (13.52)
Caucasian women	136–274 (244)	275–315 (295)	316–362 (338)	363–558 (417)	0.15–0.36 (0.23)	0.37–0.93 (0.61)	0.95–2.41 (1.55)	2.43–60.50 (6.88)
Mean age in years (SD)	31.9 (3.5)	32.1 (3.7)	32.3 (3.5)	32.4 (3.5)	32.2 (3.6)	32.1 (3.5)	32.3 (3.5)	32.2 (3.6)
Female gender	54%	54%	54%	54%	54%	54%	54%	54%
*Race*								
African-American	40%	41%	40%	40%	40%	41%	40%	40%
Caucasian	60%	59%	60%	60%	60%	59%	60%	60%
*Education level*								
High school or less	22%	23%	24%	26%	21%	24%	23%	26%
More than high school	78%	77%	76%	74%	79%	76%	77%	74%
Ever-smoker	45%	45%	45%	47%	41%	45%	50%	45%
Body mass index (kg/m^2^) (SD)	24.0 (3.7)	25.2 (4.4)	26.4 (4.9)	29.6 (7.0)	23.4 (3.4)	25.4 (4.0)	26.7 (5.0)	29.7 (7.0)

a Fibrinogen expressed in mg/dL, CRP expressed in µg/mL.

To determine whether individuals in the highest and lowest year 7 fibrinogen and CRP quartiles remained in that quartile at year 20, we cross-tabulated the earlier and year 20 quartiles to measure the percentage of those in a given quartile at year 7 who remained in that quartile at year 20. We additionally determined the Spearman rank correlation coefficient in all participants between the earlier and year 20 time-points for both fibrinogen and CRP level.

Multivariable linear regression was used to assess the associations between fibrinogen or CRP and change in lung function from the year 5 to the year 20 examination (determined by subtracting the year 20 FEV_1_ or FVC measurement from the year 5 measurement). Because it was not normally distributed, year 7 CRP was log transformed. Adjustment was made for race-sex group, age, age-squared, height, and height-squared at year 20 [19], BMI at the time of fibrinogen or CRP measurement, change in waist circumference from year 7 to year 20, cumulative pack-years of cigarette smoking, and year 5 lung function.

Generalized estimating equations (GEE) were used to examine trends in FEV_1_ and FVC as a function of age over the 15 years of study. The GEE model included the following covariates: race-sex group, exam year, study center, and time-dependent age, time-dependent height and height-squared, year 5 waist circumference and time-dependent change in waist circumference, time-dependent cumulative pack-years of cigarette exposure, and quartiles of fibrinogen or CRP. In one model, designed to provide a detailed visual assessment of goodness of fit to linear slope over age, age was categorized into 2-year age groups, adjusted mean lung function values were computed for each of the 14 age groups (starting with 23–24 year olds) for each quartile and plotted. In a second model, age was treated as a continuous variable and interactions between quartiles of fibrinogen or CRP and age were included to assess whether age trends for quartile 4 differed from quartile 1.

Logistic regression was used to assess the associations of fibrinogen or CRP with year 20 spirometry values less than 80% of the predicted value according to published race- and sex-specific equations [19], COPD using the Global Initiative for Obstructive Lung Disease (GOLD) definition of FEV_1_/FVC ≤0.70 [20], and a non-specific spirometric abnormality defined by both FEV_1_ and FVC <80% predicted with a FEV_1_/FVC >0.70, a phenotype that is not addressed in the GOLD guidelines. Adjustment was made for study center, cigarettes smoked per day and BMI at the time of fibrinogen or CRP measurement and waist circumference and cumulative pack years of exposure to cigarettes at the time of lung function measurement. Multiplicative interactions between CRP and fibrinogen and cumulative pack years of exposure to cigarettes were also determined, and when significant (p for the interaction term <0.05) stratification by smoking was performed. When analyzed as a continuous variable, CRP was log transformed to better achieve the requirement that the logit be linear in its parameters.

All statistical analyses were conducted using SAS for Windows, release 9.2 (SAS Institute Inc., Cary, NC).

## Results

### Subject characteristics

The plasma levels for the race- and sex-specific year 7 fibrinogen and CRP quartiles and demographic characteristics of the study population are included in Table 1. As expected, ever smoking and higher BMI were associated with higher fibrinogen and CRP. Because fibrinogen and CRP are both acute phase reactants, we assessed whether individuals with high fibrinogen or CRP at year 7 continued to have high levels at year 20. The majority of participants in the highest and lowest quartiles of fibrinogen or CRP at year 7 remained in that quartile at year 20 (Table 2).

**Table 2:** Spearman rank correlation coefficients between year 7 and year 20 fibrinogen and CRP measurements.*

Gender	Spearman rank correlation coefficient*	% (95% CI) in quartile 1 at year 7 also in quartile 1 at year 20	% (95% CI) in quartile 4 at year 7 also in quartile 4 at year 20
**CRP**			
Male	0.56	54 (48, 61)	51 (45, 58)
Female	0.56	55 (49, 61)	53 (47, 50)
**Fibrinogen**			
Male	0.51	53 (46, 59)	49 (42, 55)
Female	0.57	51 (45, 57)	54 (48, 60)

*P<0.001 for each correlation coefficient shown.

### Systemic inflammation in young adults and change in lung function over 15 years

Higher year 7 fibrinogen as well as CRP were associated with greater 15 year decline in both FVC and FEV_1_. (Table 3).

**Table 3:** Adjusted loss of FVC and FEV_1_ from year 5 to year 20 across race-sex specific quartiles of year 7 fibrinogen and CRP as well as linear regression coefficients for fibrinogen and log-transformed CRP as continuous variables.*

Loss of Lung Function	Fibrinogen Quartile (Year 7)					
(Year 5 minus Year 20)	1	2	3	4	Beta†	*P*
FEV_1_ (mL)	446	456	464	487	0.333	<0.001
FVC (mL)	398	14	441	439	0.418	<0.001

	CRP Quartile (Year 7)					
	1	2	3	4	Beta†	*P*
FEV_1_ (mL)	442	453	468	491	16.383	0.001
FVC (mL)	390	422	425	455	19.199	<0.001

*Adjusted for center, race-sex group, age, age-squared, height, and height-squared at year 20 [19], BMI at the time of fibrinogen or CRP measurement, change in waist circumference from year 7 to year 20, cumulative pack-years of cigarette smoking, and year 5 lung function.

† Multivariable linear regression coefficient for the prediction of change in lung function from year 5 to year 20 by year 7 fibrinogen or CRP

### Models of lung function with age and the interaction with fibrinogen and CRP

**Figure 2:**
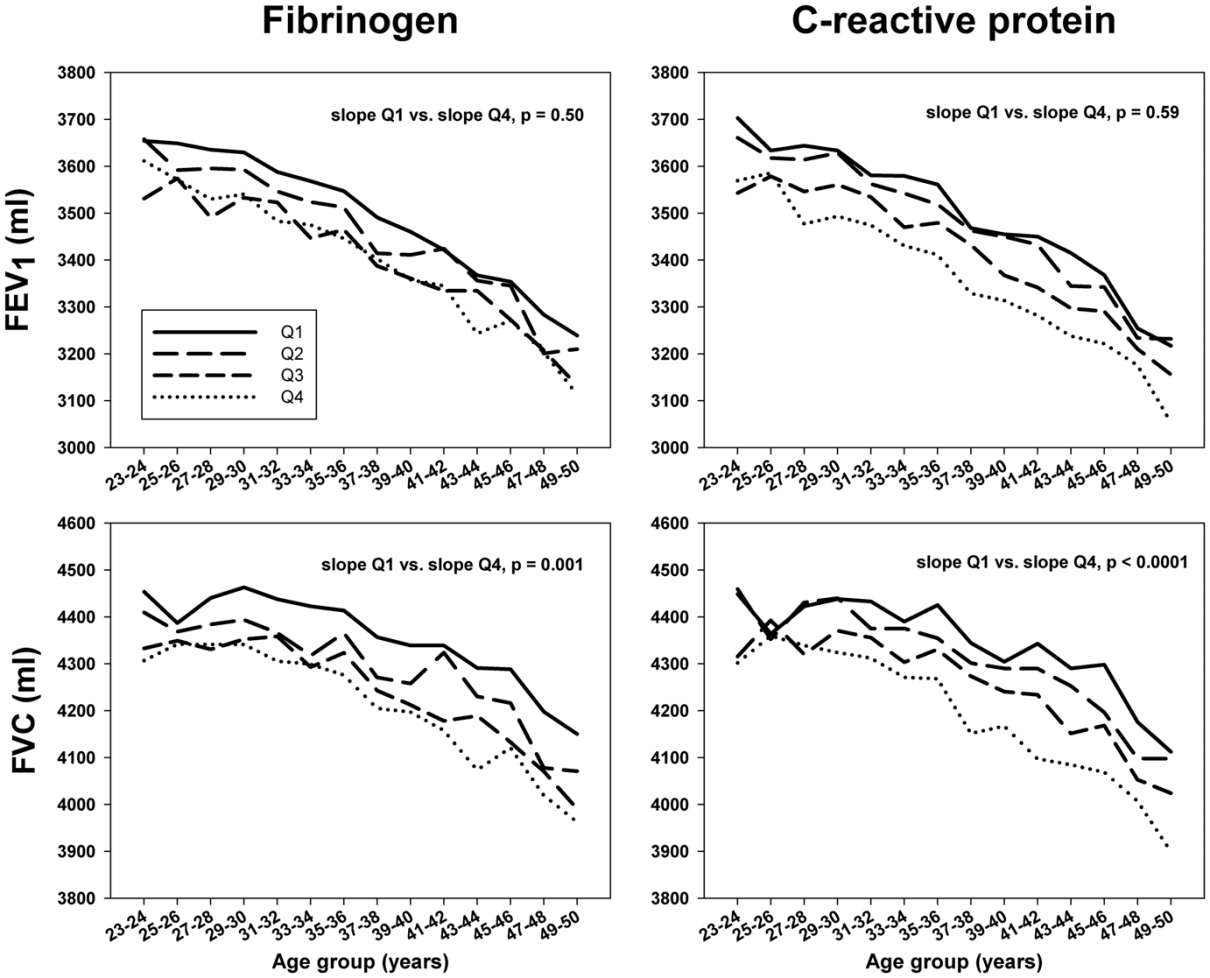
Relationship between age, lung function and quartile of fibrinogen or CRP (measured at year 7). Spirometry exams performed at CARDIA visits at years 5, 10, and 20 were used to determine a mean lung function for each 2 year age group across year 7 CRP or fibrinogen quartiles, and slopes compared between the highest and lowest quartile groups. For both fibrinogen and CRP, the age-related decline in FVC was significantly different between quartiles 1 and 4. There were no significant differences in the FEV_1_ across CRP or fibrinogen quartiles.Covariates in the model include race-sex group, exam year, pack-years of cigarette exposure, and time-dependent age, time-dependent height and height-squared, and year 5 waist circumference and time-dependent change in waist circumference.

The interaction between systemic inflammation in young adults and age-related decline in lung function was assessed using spirometry values from exam years 5, 10, and 20 in a GEE model. For both year 7 fibrinogen and CRP, quartile 4 had a greater age-related decline in FVC compared with quartile 1 (P value for the age-fibrinogen quartile or age-CRP quartile interaction on decline in lung function <0.001). In contrast, there was no significant interaction between age-related decline in FEV_1_ and fibrinogen or CRP (Figure 2).

### Systemic inflammation in young adults and abnormal lung function 13–15 years later

Year 7 fibrinogen was associated with abnormal year 20 FVC and FEV_1_, as well as the non-specific abnormality at year 20, but not COPD (Table 4). Year 7 CRP was associated with abnormal year 20 FVC, but not FEV_1_, the non-specific abnormality, or COPD (Table 4). We did not observe an interaction between fibrinogen and pack-years cigarette smoking on any outcomes. A positive interaction was observed between CRP and pack-years cigarette smoking on the COPD endpoint (but no other endpoints related to CRP) prompting subsequent analysis of the association between year 7 CRP and COPD stratified by smoking. Among participants with 10 or more pack years of cigarette smoking, higher year 7 CRP was associated with greater odds of COPD at year 20 (Table 5).

**Table 4:** Adjusted odds ratios (95% CI) for FEV_1_ or FVC less than 80% predicted, COPD using the GOLD definition (FEV_1_/FVC ≤0.70) or a non-specific spirometry abnormality (FEV_1_ and FVC <80% with FEV_1_/FVC >0.70) at year 20 across race- and sex-specific quartiles of year 7 fibrinogen and CRP and per standard deviation of fibrinogen or log-transformed CRP.*

Spirometry	Year 7 Fibrinogen Quartile					Per SD†	
(year 20)	N	1	2	3	4	(mg/dL)	*P*
FEV_1_<80%	241	1.0	1.11 (0.71, 1.73)	1.49 (0.98, 2.26)	1.23 (0.79, 1.91)	1.37 (1.19, 1.59)	<0.001
FVC<80%	214	1.0	1.27 (0.77, 2.09)	2.02 (1.28, 3.21)	1.77 (1.09, 2.87)	1.51 (1.30, 1.75)	<0.001
COPD (GOLD)	126	1.0	0.72 (0.42, 1.22)	1.00 (0.61, 1.65)	0.87 (0.50, 1.51)	0.93 (0.75, 1.16)	0.51
Non-specific abnormality	149	1.0	1.10 (0.62, 1.97)	1.78 (1.05, 3.01)	1.27 (0.77, 2.23)	1.47 (1.19, 1.68)	<0.001

	Year 7 CRP Quartile					Per SD†	
	N	1	2	3	4	(µg/mL)	*P*
FEV_1_ <80%	241	1.0	0.79 (0.51, 1.22)	0.94 (0.62, 1.43)	0.98 (0.63, 1.51)	1.17 (1.00, 1.37)	0.05
FVC <80%	214	1.0	0.89 (0.55, 1.44)	1.26 (0.80, 1.99)	1.36 (0.85, 2.17)	1.35 (1.14, 1.59)	<0.001
COPD (GOLD)	126	1.0	0.94 (0.56, 1.57)	0.90 (0.53, 1.54)	1.13 (0.66, 1.96)	0.98‡ (0.80, 1.21)	0.86
Non-specific abnormality	149	1.0	0.70 (0.41, 1.20)	0.82 (0.49, 1.39)	0.81 (0.47, 1.40)	1.14 (0.94, 1.39)	0.18

*Adjusted for center, cigarettes/d, BMI at the time of fibrinogen and CRP measurement, and waist circumference and cumulative pack-years of cigarette exposure at the time of spirometry.

† Adjusted odds ratio per standard deviation of year 7 fibrinogen or log-transformed CRP.

‡ Test for CRP x pack-years cigarette exposure interaction significant (*P* = 0.005).

**Table 5:** Adjusted odds ratios (95% CI) for COPD using the GOLD definition (FEV_1_/FVC ≤0.70) at year 20 across race- and sex-specific quartiles of year 7 CRP and per standard deviation of CRP stratified by pack-years cigarette exposure.*

Pack-Years	Year 7 CRP Quartile					Per SD†	
Smoking	N‡	1	2	3	4	(µg/mL)	*P*
0 pack-years (N = 1249)	53	1.0	0.66 (0.32, 1.38)	0.48 (0.21, 1.12)	0.72 (0.32, 1.60)	0.77 (0.55, 1.06)	0.11
1–9 pack-years (N = 501)	26	1.0	1.11 (0.43, 2.88)	0.29 (0.06, 1.42)	0.95 (0.26, 3.52)	0.78 (0.46, 1.32)	0.35
10+ pack-years (N = 382)	47	1.0	2.46 (0.60, 10.11)	5.01 (1.38, 18.22)	4.90 (1.29, 18.59)	1.53 (1.08, 2.16)	0.02

*Adjusted for center, cigarettes/day, and BMI at the time of CRP measurement, and waist circumference and cumulative pack-years of cigarette exposure at the time of spirometry.

† Adjusted odds ratio for COPD per standard deviation of log-transformed year 7 CRP.

‡ Number of year 20 COPD cases in each smoking stratum.

To evaluate the effects of systemic inflammation independent of baseline lung function, we repeated this analysis excluding individuals with abnormal lung function at year 5: 87 participants had abnormal year 5 FEV_1_, 53 had abnormal year 5 FVC, 52 had COPD at year 5, and 40 had the non-specific abnormality at year 5 and were therefore excluded. The association between year 7 fibrinogen and year 20 FEV_1_ <80% predicted was preserved (odds ratio (OR) per standard deviation fibrinogen 1.38 (95% confidence interval (CI): 1.17–1.63; *P*<0.001) as was the association with year 20 FVC <80% predicted (OR per standard deviation fibrinogen 1.55 (95% CI: 1.31–1.83, *P*<0.001) and the non-specific spirometry abnormality at year 20 (OR per standard deviation fibrinogen 1.50 (95% CI: 1.16–1.70; *P*<0.001). In addition, the association year 7 CRP with FVC <80% predicted at year 20 remained significant (OR per standard deviation log-transformed CRP 1.33 (95% CI: 1.11–1.59; *P* = 0.002). The positive interaction between pack-years of cigarette exposure and CRP on the COPD endpoint remained significant (*P* for the log transformed CRP x pack-years exposure interaction = 0.04). We also repeated the analysis of CRP and COPD stratified by smoking excluding those with COPD at baseline. While no longer statistically significant, the trend for the association between CRP and COPD remained among participants with 10 or more pack-years (OR per standard deviation log transformed CRP 1.36 (95% CI: 0.91, 2.03, *P* = 0.14)).

## Discussion

We report three major findings. First, greater systemic inflammation in young adults is associated with greater decline in lung function over 15 years as well as having abnormal lung function 13 years later. Participants in the highest year 7 fibrinogen or CRP quartiles had greater FEV_1_ and FVC decline compared to those with the lowest quartiles over 15 years of follow-up. Second, higher levels of year 7 fibrinogen and CRP in young adulthood are associated with greater risk of having an abnormal FVC (fibrinogen and CRP) and FEV_1_ (fibrinogen) in middle-age. Third, among participants who smoked 10 or more pack-years of cigarettes, year 7 CRP is associated with increased risk of having obstructive lung disease at year 20.

Cross-sectional studies have documented that increased systemic inflammation is associated with lower lung function in both healthy adults and patients with COPD [1], [2], [3], [21]. In older populations, there appears to be an association between systemic inflammation and risk of COPD. In a Dutch study of subjects 55 years of age and older, CRP was associated with increased risk of incident COPD [6], and in a study of 5,247 healthy Swedish men of mean age 46 years, a panel of inflammation sensitive plasma proteins including CRP was associated with increased risk of COPD hospitalization [5]. There are, however, limited and conflicting reports of the association between systemic inflammation and lung function change over time in young adults. Fogarty, et al found no association between baseline CRP and decline in lung function over nine years in 2,442 individuals aged 18–70 years [8]. Among 531 participants 20 to 44 years of age in the European Community Respiratory Health Survey, increases in CRP over an 8 year period were associated with greater annual declines in FEV_1_, although there was no significant association between baseline CRP and annual decline in FEV_1_ [7]. We performed a previous analysis from CARDIA that found that participants in the highest quartile of fibrinogen at year 5 of the study had year 10 FEV_1_ and FVC values that were 45 ml and 67 ml lower than those in the lowest quartile [9]. We now extend upon this prior work to document that both fibrinogen and CRP in young adults are associated with accelerated decline in lung function as well as abnormal lung function over 15 years of followup.

The origins of elevated markers of systemic inflammation in the CARDIA cohort are uncertain. One can speculate that early lung inflammation resulting from environmental exposures beyond cigarette smoking results in decreasing lung function over time. These exposures could also be accompanied by increased levels of plasma fibrinogen and CRP as the systemic response to the primary locus of inflammation in the lung. Both cigarette smoke and ambient particulate matter have a high burden of oxidant molecules. When inhaled, these likely result in significant local oxidant stress in the lung resulting in local increases in proinflammatory gene expression [22]. Ambient particulate matter has been shown to stimulate release of TNF-α as well as other cytolines including granulocyte macrophage colony-stimulated factor (GM-CSF), interleukin (IL)-6 and IL-1β by human alveolar macrophages [23]. These cytokines may then “spill-over” into the systemic circulation where they induce production of acute phase reactants such as fibrinogen and CRP. The absence of samples collected directly from the lungs of CARDIA participants which could then be correlated with CRP and fibrinogen in the blood limits our ability to explore this possibility further in the CARDIA cohort.

Alternatively, some individuals may have genetic variation that results in high systemic inflammation which results in increased risk of adverse health outcomes if they have the additional exposure of cigarette smoking [24], [25]. These individuals may have an “inflammatory phenotype” characterized by concurrent exaggerated inflammatory responses in both the lung and systemic compartments. We observed that among participants at the highest risk for developing COPD (10 or more pack-years of smoking cigarettes), those with higher CRP in young adulthood had elevated risk of COPD in middle age. This indicates that smoking in a vulnerable population (those with high CRP) might confer an accelerated risk of obstructive lung disease, compared to the elevated inflammatory marker alone. Regardless of whether CRP elevation is the result of “spill-over” or reflective of an “inflammatory phenotype”, it may be valuable as a marker to identify those cigarette smokers who are at greatest risk of developing COPD in the future.

In our study, CRP and fibrinogen were not consistently associated with the same patterns of lung function change over time. We found that year 7 fibrinogen and CRP were both associated with low FVC at year 20, but fibrinogen was also associated with abnormal FEV_1_ and the non-specific reduction in both FEV_1_ and FVC with a preserved FEV_1_/FVC. In addition, while there was an interaction between CRP and cigarette smoking, fibrinogen was not associated with COPD and did not interact with smoking. Fibrinogen, therefore, may be a marker of individuals at risk for restrictive lung disease. Alternatively, in non-smokers, high fibrinogen and CRP may mark risk for accelerated age-related decline in lung function, without the development of any specific lung disease. Both patterns and their respective blood markers are of considerable interest since reductions in both FEV_1_ and FVC in the absence of a diagnosis of lung disease are associated with cardiovascular and metabolic disease [3], [26].

It has recently been advocated that COPD be incorporated into the “chronic systemic inflammatory syndrome” [27]. This syndrome-based approach relies on the concept that chronic systemic inflammation underlies not only COPD, but also other co-morbid conditions, including cardiovascular disease and the metabolic syndrome [27]. Our data indicate that systemic inflammation in young adults, particularly CRP in cigarette smokers, is associated with future impairment in lung health, prompting the hypothesis that the systemic inflammation that links COPD with various co-morbidities may be detectable prior to the onset of clinically overt disease.

The major strength of our study is the long-term followup of a cohort with serial lung function measurements. In addition, the age of the cohort at the year 20 examination allows us to begin to evaluate whether accelerated decline in lung function that occurs in association with high levels of systemic inflammation in young adults which has been reported by others [10], and confirmed in our present report, actually results in clinical disease. We document that in the highest risk group (those with 10 or greater pack years of cigarette smoking) CRP in young adults is associated with the presence of COPD 13 years later.

We note some limitations in our study. CRP and fibrinogen measurements were not measured at the baseline visit. However, the relatively early measurement of fibrinogen and CRP (year 7) compared to lung function measurement (year 20) ensures that the exposure of systemic inflammation predated, by more than a decade, our principle outcome measurements. While both fibrinogen and CRP are acute phase reactants and the duration of exposure can be called into question, we documented that those in the highest and lowest quartiles at the early time point of measurement tend to remain in those categories at later time points. The absence of specimens that directly measure lung inflammation in the cohort limits our ability to link the inflammatory responses in the systemic and respiratory compartments. Another limitation is the absence of post-bronchodilator spirometry testing as well as a lack of information about static lung volumes or lung imaging, which would provide insights into the structural changes in the lungs of participants observed to have physiologic abnormalities. In addition, although we have adjusted for waist circumference at the time of lung function measurement and BMI at the time of fibrinogen and CRP measurement in all statistical models, our analysis remains vulnerable to residual confounding due to possible associations between obesity, lung function, and inflammation. Since both increased levels of CRP and fibrinogen were associated at baseline with increased BMI, the possibility that obesity plays a critical role in the lung function–systemic inflammation relationship remains a clear possibility. Indeed, obesity is associated with high CRP and fibrinogen [28] and lower lung function [29] and may provide another link between systemic inflammation and low lung function.

In conclusion, among a generally healthy population of young adults, higher levels of plasma fibrinogen and CRP are associated with accelerated decline in lung function. In individuals who smoke more than 10 pack-years of cigarettes, higher levels of CRP during young adulthood are associated with greater risk of COPD in middle age. These findings indicate that individuals with increased systemic inflammation early in life may be at increased risk for future impairments in lung health, and CRP, in particular, may be a useful marker of vulnerability for COPD among cigarette smokers.

## References

[1] Hancox RJ, Poulton R, Greene JM, Filsell S, McLachlan CR (2007). Systemic inflammation and lung function in young adults.. Thorax.

[2] Aronson D, Roterman I, Yigla M, Kerner A, Avizohar O (2006). Inverse Association between Pulmonary Function and C-Reactive Protein in Apparently Healthy Subjects.. Am J Respir Crit Care Med.

[3] Mannino DM, Ford ES, Redd SC (2003). Obstructive and restrictive lung disease and markers of inflammation: data from the Third National Health and Nutrition Examination.. Am J Med.

[4] Walter RE, Wilk JB, Larson MG, Vasan RS, Keaney JF (2008). Systemic Inflammation and COPD: The Framingham Heart Study.. Chest.

[5] Engstrom G, Segelstorm N, Ekberg-Aronsson M, Nilsson PM, Lindgarde F (2009). Plasma markers of inflammation and incidence of hospitalisations for COPD: results from a population-based cohort study.. Thorax.

[6] van Durme YMTA, Verhamme KMC, Aarnoudse A-JLHJ, Van Pottelberge GR, Hofman A (2009). C-Reactive Protein Levels, Haplotypes, and the Risk of Incident Chronic Obstructive Pulmonary Disease.. Am J Respir Crit Care Med.

[7] Shaaban R, Kony S, Driss F, Leynaert B, Soussan D (2006). Change in C-reactive protein levels and FEV1 decline: a longitudinal population-based study.. Respir Med.

[8] Fogarty AW, Jones S, Britton JR, Lewis SA, McKeever TM (2007). Systemic inflammation and decline in lung function in a general population: a prospective study.. Thorax.

[9] Thyagarajan B, Jacobs DR, Apostol GG, Smith LJ, Lewis CE (2006). Plasma fibrinogen and lung function: the CARDIA Study.. Int J Epidemiol.

[10] Rasmussen F, Mikkelsen D, Hancox RJ, Lambrechtsen J, Nybo M (2009). High-sensitive C-reactive protein is associated with reduced lung function in young adults.. Eur Respir J.

[11] Hughes GH, Cutter G, Donahue R, Friedman GD, Hulley S (1987). Recruitment in the Coronary Artery Disease Risk Development in Young Adults (CARDIA) Study.. Control Clin Trials.

[12] Friedman GD, Cutter GR, Donahue RP, Hughes GH, Hulley SB (1988). CARDIA: study design, recruitment, and some characteristics of the examined subjects.. J Clin Epidemiol.

[13] Reiner AP, Carty CL, Carlson CS, Wan JY, Rieder MJ (2006). Association between patterns of nucleotide variation across the three fibrinogen genes and plasma fibrinogen levels: the Coronary Artery Risk Development in Young Adults (CARDIA) study.. J Thromb Haemost.

[14] Lakoski SG, Herrington DM, Siscovick DM, Hulley SB (2006). C-reactive protein concentration and incident hypertension in young adults: the CARDIA study.. Arch Intern Med.

[15] American Thoracic Society (1979). Snowbird workshop on standardization of spirometry.. Am Rev Respir Dis.

[16] American Thoracic Society (1987). Standardization of spirometry—1987 update.. Am Rev Respir Dis.

[17] American Thoracic Society (1995). Standardization of Spirometry, 1994 Update.. Am J Respir Crit Care Med.

[18] Miller MR, Hankinson J, Brusasco V, Burgos F, Casaburi R (2005). Standardisation of spirometry.. Eur Respir J.

[19] Hankinson JL, Odencrantz JR, Fedan KB (1999). Spirometric reference values from a sample of the general U.S. population.. Am J Respir Crit Care Med.

[20] Rabe KF, Hurd S, Anzueto A, Barnes PJ, Buist SA (2007). Global strategy for the diagnosis, management, and prevention of chronic obstructive pulmonary disease: GOLD executive summary.. Am J Respir Crit Care Med.

[21] Pinto-Plata VM, Mullerova H, Toso JF, Feudjo-Tepie M, Soriano JB (2006). C-reactive protein in patients with COPD, control smokers and non-smokers.. Thorax.

[22] MacNee W (2005). Pulmonary and systemic oxidant/antioxidant imbalance in chronic obstructive pulmonary disease.. Proc Am Thorac Soc.

[23] van Eeden SF, Tan WC, Suwa T, Mukae H, Terashima T (2001). Cytokines involved in the systemic inflammatory response induced by exposure to particulate matter air pollutants (PM(10)).. Am J Respir Crit Care Med.

[24] Pankow JS, Folsom AR, Cushman M, Borecki IB, Hopkins PN (2001). Familial and genetic determinants of systemic markers of inflammation: the NHLBI family heart study.. Atherosclerosis.

[25] Sunyer J, Pistelli R, Plana E, Andreani M, Baldari F (2008). Systemic inflammation, genetic susceptibility and lung function.. Eur Respir J.

[26] Mannino DM, Thorn D, Swensen A, Holguin F (2008). Prevalence and outcomes of diabetes, hypertension and cardiovascular disease in COPD.. Eur Respir J.

[27] Fabbri LM, Rabe KF (2007). From COPD to chronic systemic inflammatory syndrome?. Lancet.

[28] Haffner SM (2007). Abdominal Adiposity and Cardiometabolic Risk: Do We Have All the Answers?. Am J Med.

[29] Rossi A, Fantin F, Di Francesco V, Guariento S, Giuliano K (2008). Body composition and pulmonary function in the elderly: a 7-year longitudinal study.. Int J Obes.

