# Correction: Systemic Inflammation in Young Adults Is Associated with Abnormal Lung Function in Middle Age

**DOI:** 10.1371/annotation/ed92662b-c566-488d-b090-6c80046d3499

**Published:** 2010-08-06

**Authors:** Ravi Kalhan, Betty T. Tran, Laura A. Colangelo, Sharon R. Rosenberg, Kiang Liu, Bharat Thyagarajan, David R. Jacobs, Lewis J. Smith

In Table 3, the FVC loss for quartile 2 should be "414 mL" rather than "14 mL."

